# A Comparison of Modern and Popular Approaches to Calculating Reliability for Dichotomously Scored Items

**DOI:** 10.1177/01466216221084210

**Published:** 2022-04-14

**Authors:** Sébastien Béland, Carl F. Falk

**Affiliations:** 1 Administration et fondements de l'éducation, 5622 Université de Montréal, QC, Canada; 2 Department of Psychology, 5620 McGill University, Montréal, QC, Canada

**Keywords:** reliability, factor analysis, item response theory, classical test theory, dichotomous data

## Abstract

Recent work on reliability coefficients has largely focused on continuous items, including critiques of Cronbach’s alpha. Although two new model-based reliability coefficients have been proposed for dichotomous items (Dimitrov, 2003a,b; Green & Yang, 2009a), these approaches have yet to be compared to each other or other popular estimates of reliability such as omega, alpha, and the greatest lower bound. We seek computational improvements to one of these model-based reliability coefficients and, in addition, conduct initial Monte Carlo simulations to compare coefficients using dichotomous data. Our results suggest that such improvements to the model-based approach are warranted, while model-based approaches were generally superior.

## Introduction

Reliability is a fundamental element of scientific measurement (Brennan, 2001; Klaassen & van Peppen, 1986). In psychometric applications, reliability is sometimes described as a way to quantify measurement error: “a unitless index assigned to a measurement instrument, taking its value on the interval [0, 1], for the extent to which the measurement instrument is free from error, with the values 0 and 1 corresponding to the extreme cases of ‘pure error’ and ‘no error,’ respectively” (Li, 2003, p. 91).

There are traditionally three strategies to operationalize reliability; the first two implicate the idea of reliability across time (called test-retest procedure) and across alternative versions of the same test. In this article, we consider the internal consistency strategy, which requires only a single administration of the test and of which alpha ( α hereafter; Cronbach, 1951) is a classic example. We propose that the internal consistency approach is the most widely used in educational and psychological measurement.

The proper assessment of the reliability of items from a dichotomous test (e.g., correct and incorrect answers) has been neglected: "the majority of the work on it has been concerned with reliability of multiple-item measuring instruments consisting of continuous components” (Raykov et al. (2010, p. 265). Indeed, many authors have recommended against α  as a measure of reliability (e.g., cf. Savalei & Reise, 2019; Sijtsma, 2009; McNeish, 2018; Raykov & Marcoulides, 2019), with some suggesting alternatives such as omega coefficients (McDonald, 1985, 1999) and the greatest lower bound (Jackson & Agunwamba, 1977). These articles are largely based on research amongst continuous items.

While many early studies of reliability were rooted in classical test theory (*CTT*), several researchers have discussed reliability for dichotomous items from a model-based perspective. Bartholomew and colleagues (Bartholomew et al., 1993) defined a reliability coefficient based in part on the two-parameter logistic model (*2PLM*, Birnbaum, 1968), which may be thought of as a non-linear factor analysis model for dichotomous data (Bartholomew et al., 2008). The 2PLM is one item model under a broader item response theory (IRT) framework, which usually views reliability as conditional upon the level of the latent construct. Bartholomew and colleagues’ work instead allowed computation of a single reliability coefficient as an overall property of test scores. A single reliability coefficient derived from an IRT model allows applied researchers the convenience of reporting a single coefficient, and is in a similar vein as prior work on marginal reliability (Green, Bock, Humphreys, Linn, & Reckase, 1984).

While Bartholomew et al.’s work assumed the researcher created a weighted sum of item scores, it may be more common to compute a test score composite as an unweighted sum. Dimitrov (2003a, 2003b) presented a strategy to define and estimate a single reliability coefficient under these conditions while using the 2PLM. The total score (also referred to as the test score composite, sum score, or number correct score) when computed as an unweighted sum is defined as the following

$$Y=Y_{1}+Y_{2}+\cdots +Y_{I}$$

where $Y_{1}$ through $Y_{I}$ are dichotomous responses (scored 0 and 1) to *I* items*.* This latter case is appropriate when the total of correctly answered items is used as the test score on a multiple choice exam. Although use of this sum score differs from typical IRT or factor analytic scoring methods, this manuscript mainly concerns the reliability of this scoring approach. We argue that this is the most ubiquitous type of data in educational testing, and is common on some psychological tests^1^ (e.g., Raskin & Hall, 1979).

The goal of Dimitrov (2003a, 2003b) was to define and estimate reliability with its classical definition, defined as a ratio of true score variance to total variance

(1) $$\rho_{yy}=\frac{Var\left(T\right)}{Var\left(Y\right)}=\frac{Var\left(T\right)}{Var\left(T\right)+Var\left(E\right)}=1-\frac{Var\left(E\right)}{Var\left(Y\right)}$$

where the variance of the total score is decomposed into true score variance, Var(T) , and error variance, Var(E) , since Var(Y)=Var(T)+Var(E) (Lord & Novick, 1968). To further develop this method and make it accessible, Raykov et al. (2010) proposed a strategy to both compute confidence intervals for reliability and estimates of change in reliability when an item is deleted, also using item parameters from the 2PLM. This reliability estimate is based on a function of parameters and standard errors using the Delta method and was implemented with M*plus* (Muthén & Muthén, 1998-2017). Given the equivalence of factor analysis of categorical items and some item response theory models (e.g., Kamata & Bauer, 2008; Takane & de Leeuw, 1987), it may not be surprising that a similar strategy for estimating reliability was developed by Green and Yang (2009b) for nonlinear factor analysis models. This latter approach may very well be equivalent to that of Raykov et al. (2010), but small differences in reliability estimates may occur if the model is estimated in a different way (e.g., Bolt, 2005).

Given these recent developments and the continued use of overall reliability coefficients, including for dichotomous items, more advice is required for applied researchers who may implement such a coefficient. What evidence is there that either approach by Dimitrov (2003a, 2003b) or Green & Yang (2009b) performs better relative to each other, versus Cronbach’s *α*, model-based reliability coefficients from linear structural equation modeling, or any other host of coefficients that could be obtained from the literature (e.g., Guttman’s lambda or the great lower bound (*GLB*))? Dimitrov (2003a, 2003b) and Raykov et al. (2010) do not present results of simulations, although there are explicit comparisons with α in empirical examples, with α yielding lower estimates of reliability, possibly due to bias and underestimation. Green & Yang (2009b) conducted a small set of simulations in which they suggest that their approach is superior to model-based approaches from linear structural equation modeling, and to *α*. To our knowledge, other recent comparisons with dichotomous items tend to focus exclusively on empirical data (e.g. Deng & Chan, 2017; Napolitano et al., 2013). We are therefore unaware of any study that focuses specifically on dichotomous data that has compared these recently developed model-based approaches to each other, or to any other popular alternatives.

Our main goal is to compare the aforementioned coefficients, with a focus on additionally improving Dimitrov’s (2003a, 2003b) reliability method. We note that Dimitrov’s approach, along with that of Green & Yang (2009b) have not been previously compared under idealized conditions (i.e., a correctly specified model). In the remainder of this article, we present two simulation studies: Study 1 investigates the accuracy of the Dimitrov (2003a, 2003b) analytic approach versus a quadrature-based approach for computing classical reliability from 2PLM item parameters and a known population model. Study 2 then employs simulations to compare the precision of this coefficient with that of Green and Yang, and other popular CTT-based and model-based reliability coefficients. This study is interesting in part because we study the behavior of reliability coefficients with dichotomous data sets as opposed to generating coefficient values from factor analysis model for continuous data (e.g., Trizano-Hermosilla & Alvarado, 2016).

### Study 1

In studying the accuracy of reliability estimates under the case of dichotomous items, we first need a viable way to define the true, or population-level reliability in a classical sense. Dimitrov (2003a, 2003b; see also May & Nicewander, 1994), provides both the underlying analytical formulas and computational approach for the true reliability of total scores whose item responses are generated by a 2PLM for all *I* items ( i=1,2,…,I )

(2) $$P_{i}\left(\theta \right)=\frac{exp\left[Da_{i}\left(\theta -b_{i}\right)\right]}{1+exp\left[Da_{i}\left(\theta -b_{i}\right)\right]}$$

with D equal to the scaling constant that puts the slope, $a_{i}$ , and difficulty, $b_{i}$ , on the approximate same scale as the normal ogive variant of the model (Lord & Novick, 1968). Conceptually, $P_{i}\left(\theta \right)$ provides the probability of obtaining a score of “1” on the item, conditional on the latent trait, θ . To reiterate the analytical expressions for computation of reliability, the error variance for item *i* is the following

(3) $$Var\left(E_{i}\right)=\int_{-\infty }^{\infty }P_{i}\left(\theta \right)\left[1-P_{i}\left(\theta \right)\right]\phi \left(\theta \right)d\theta$$

where the integral is across the latent distribution with ϕ(⋅) its density function. Accumulating over *I* items, the entire error variance of the test is the following

(4) $$Var\left(E\right)=\sum_{i=1}^{I}Var\left(E_{i}\right)$$

The total true-score variance of the test is the following

(5) $$Var\left(T\right)={\int_{-\infty }^{\infty }\left[\sum_{i=1}^{I}P_{i}\left(\theta \right)\right]}^{2}\phi \left(\theta \right)d\theta -{\left[\int_{-\infty }^{\infty }\sum_{i=1}^{I}P_{i}\left(\theta \right)\phi \left(\theta \right)d\theta \right]}^{2}$$

where $P_{i}\left(\theta \right)$ is the conditional probability of a correct answer on item *i*. Finally, Equations (4) and (5) can then be used to define the classic reliability of the test, $\rho_{yy}$ , via equation (1). Implicit in this approach is that the true score variability is due entirely to the underlying latent trait, θ , and any remaining item-specific variance is considered error.

As opposed to evaluating the integrals in 3 and 5 directly, Dimitrov (2003a, 2003b) proposed an analytical approximation based in part on the error function, we call “erf (.)” (see Hastings, 1955). It is claimed that the error function “can be evaluated with an absolute error smaller than 0.0005” (Dimitrov, 2003a; p. 443) to reproduce the marginal probabilities of response to any particular item while assuming a known normal population distribution for the latent trait.

Equations utilizing this approach were then developed to allow the calculation of true reliability. For instance, the marginal probability of a “1” response to item i is $P_{i}\left(\theta \right)$ weighted by the density of the latent trait distribution, and then integrating over θ

(6) $$\pi_{i}=\int_{-\infty }^{\infty }P_{i}\left(\theta \right)\phi \left(\theta \right)d\theta$$

Under the 2PLM, this quantity can also be approximated using the error function

(7) $$\pi_{i}\approx \frac{1-erf\left(Z_{i}\right)\;\;}{2}$$

where $Z_{i}=\frac{a_{i}b_{i}}{\sqrt{2\left(1+a_{i}^{2}\right)}}$ and $erf\left(Z_{i}\right)=1-{\left(1+m_{1}Z_{i}+m_{2}Z_{i}^{2}+m_{3}Z_{i}^{3}+m_{4}Z_{i}^{4}\right)}^{-4}$ assuming $m_{1}$ =0.278393, $m_{2}=$ 0.230389, $m_{3}=$ 0.000972, and $m_{4}=0.078108.\;$ In addition, when $Z_{i}$ < 0, $erf\left(-Z_{i}\right)=-erf\left(Z_{i}\right)$ .

Then, the error variance for a particular item can also be approximated by

(8) $$Var\left(E_{i}\right)\approx m_{i}exp\left[-0.5{\left(\frac{b_{i}}{d_{i}}\right)}^{2}\right]\;$$

where $m_{i}=0.2646-0.118a_{i}+0.0187a_{i}^{2}$ and $d_{i}=0.7427+0.7081/a_{i}+0.0074/a_{i}^{2}$ .

Both of these quantities can be used to approximate the true score variance for the number-right score,

(9) $$Var\left(T\right)\approx \sum_{i=1}^{I}\sum_{j=1}^{J}\sqrt{\left[\pi_{i}\left(1-\pi_{i}\right)-Var\left(E_{i}\right)\right]\left[\pi_{j}\left(1-\pi_{j}\right)-Var\left(E_{j}\right)\right]}$$

the error variance of the test, and finally the reliability of the test, $\rho_{yy}$ , using (1).

Although this technique may be computationally fast, it has not been evaluated against a more accurate approach. For example, it is not too difficult to evaluate the integral in (3) directly using rectangular quadrature

(10) $$Var\left(E_{i}\right)\approx \sum_{q=1}^{Q}P_{i}\left(X_{q}\right)\left[1-P_{i}\left(X_{q}\right)\right]W_{q}$$

where $W_{q}=\phi \left(X_{q}\right)/\sum_{q=1}^{Q}\phi \left(X_{q}\right)$ are normalized quadrature weights. The integrals in (5) can also be evaluated in this way

(11) $$Var\left(T\right)\approx {\sum_{q=1}^{Q}\left[\sum_{i=1}^{I}P_{i}\left(X_{q}\right)\right]}^{2}W_{q}-{\left[\sum_{q=1}^{Q}\sum_{i=1}^{I}P_{i}\left(X_{q}\right)W_{q}\right]}^{2}$$

If greater precision is desired, the number of weights or their spacing can be adjusted. A strategy used in this Study was to use 101 equally spaced quadrature nodes between -6 and 6.

Evaluation of any discrepancies between what we will call the *analytical-based* approach of Dimitrov (2003a, 2003b) and this *quadrature-based* approach is important, since either approach can be used in practice along with item parameters ( $a_{i}$ and $b_{i}$ for all items) to define population reliability, or with estimated parameters as a model-based estimate of reliability. Important to note, both of these approaches make the same assumptions and differ only in the computation approach.

## Method

We applied a simulation-based approach to study the accuracy of the analytic and quadrature-based approaches for computing classical reliability from 2PLM item parameters. Since true reliability is not known, and must be approximated by either approach, we instead describe the logic of an alternative criterion. Suppose that two variables, *X* and *Y*, are error-prone measures of $\delta_{X}$ and $\delta_{Y}$ , respectively. What we desire is the true correlation between these variables, $r_{\delta_{X}\delta_{Y}}$ , but can only observe the correlation between the error-prone variables, $r_{xy}$ . The well-known disattenuation formula is the following (Spearman, 1904)

(12) $$r_{\delta_{X}\delta_{Y}}=\frac{r_{xy}}{\sqrt{\rho_{xx}\rho_{yy}}}$$

which allows us to calculate the true correlation, $r_{\delta_{X}\delta_{Y}}$ , by adjusting $r_{xy}$ for the reliability of sum scores *X* and *Y*, which we denote $\rho_{xx}$ and $\rho_{yy}$ , respectively.

Now, suppose that we can simulate both error-free, $\delta_{X}$ and $\delta_{Y}$ , and error-prone sum score variables, *X* and *Y*, such that the true correlation $r_{\delta_{X}\delta_{Y}}$ is known, but the reliabilities of the individual variables, $\rho_{xx}$ and $\rho_{yy}$ , are not known. Based on such data, it should be possible to evaluate the accuracy of different ways of computing $\rho_{xx}$ and $\rho_{yy}$ . Specifically, along with an estimate of $\;r_{xy}$ , a method that more accurately computes $\rho_{xx}$ and $\rho_{yy}$ should allow better recovery of the true correlation, $r_{\delta_{X}\delta_{Y}}$ , when computed using (12).

The general strategy for this study was to simulate latent variables, $\delta_{X}$ and $\delta_{Y}$ , with both unit variance and from a multivariate normal distribution with a known population correlation. These represent error-free versions of two variables. Based on a set of items for each latent variable, and a set of known item parameters for the 2PLM, item responses to *I* items per latent construct ( $X_{1},\ldots ,X_{I}$ and $Y_{1},\ldots ,Y_{I}$ ) were then generated for each true value of the latent variable. Error-prone versions of $\delta_{X}$ and $\delta_{Y}$ were then constructed based on the idea of sum score, respectively: $X=\sum_{i=1}^{I}X_{i}$ and $Y={\sum_{i=1}^{I}Y}_{i}$ . The final step then involved calculating an estimate of the error-prone correlation between X and Y , ${\hat{r}}_{xy}$ , and then adjusting this correlation estimate by the reliabilities, $\rho_{xx}$ and $\rho_{yy}$ , to arrive at an estimate of ${\hat{r}}_{\delta_{X}\delta_{Y}}$ . Since the true item parameters are known, we directly computed the reliability of the sum scores using either quadrature or analytical approximations.

Study 1 was a 3 (correlation method: ${\hat{r}}_{\delta_{X}\delta_{Y}}$ with reliabilities based on either quadrature or analytical formulas, and ${\hat{r}}_{xy}$ ) × 4 (true correlation, $r_{\delta_{X}\delta_{Y}}=\;.3,.5,.7,.9$ ) × 13 (number of items per construct: 10 to 70 in increments of five) factorial design. Each combination of true correlation with their specific number of items, was generated; that being 5000 datasets with *N* = 500 respondents each. All three correlation estimates were then computed and recorded for each dataset. Latent trait scores were generated using the mvrnorm function in the MASS package (Venables & Ripley, 2002). True item parameters were drawn randomly across items and datasets, with $a_{i}∼logN\left(-.75,.25^{2}\right)$ and $b_{i}∼N\left(0,.75^{2}\right)$ . Item responses were generated using standard techniques for the 2PLM (we used the cacIRT package; Lathrop, 2015).

## Results and Discussion

**Figure 1.**
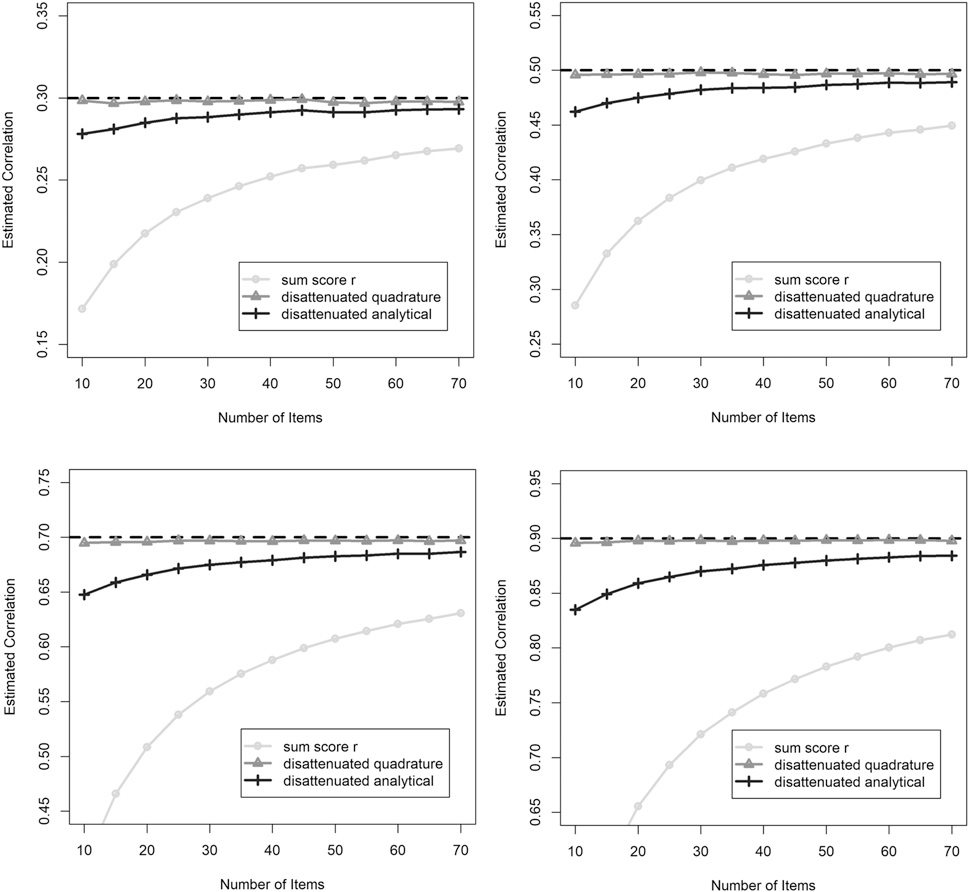
Estimated correlation where the true correlation is $r_{\delta_{X}\delta_{Y}}=\;.3$ (top-left), $r_{\delta_{X}\delta_{Y}}=\;.5$ (top-right), $r_{\delta_{X}\delta_{Y}}=\;.7$ (bottom-left) and $r_{\delta_{X}\delta_{Y}}=\;.9$ (bottom-right).

In this segment, we report the average correlation estimates across all 5000 datasets in each cell of the experimental design. We graphically depict these correlations for the full range of number of items per construct, for each of the three correlation estimation methods, and for all true correlation values (Figure 1 top-left: $r_{\delta_{X}\delta_{Y}}=\;.3$ , top-right: $r_{\delta_{X}\delta_{Y}}=\;.5$ , bottom-left: $r_{\delta_{X}\delta_{Y}}=\;.7$ , and bottom-right: $r_{\delta_{X}\delta_{Y}}=\;.9$ ).

The true correlation is represented as a horizontal dashed line. The sum score correlation was error-prone and systematically underestimated the true correlation, but improved as the number of items (and therefore reliability) increased. Disattenuated correlations using the analytical approximation for reliability also resulted in an underestimate of the true correlation, but also improved as the number of items increased. For example, in the case where the true correlation was .7 and the number of items was 10 per construct, the average estimated correlation for this method was approximately .65. The quadrature-based approximation was barely affected by the number of items, and resulted in a milder underestimate of the true correlation. This method never resulted in an average disattenuated correlation that differed from the true correlation by more than .006 in absolute value.

Since smaller values of reliability in the disattenuation formula tend to result in larger adjusted correlations, this pattern of results suggests that the analytical approximation would produce a mild overestimate of true reliability. The values of reliability, $\rho_{xx}$ and $\rho_{yy}$ , produced by the analytical approximation, are too large to fully disattenuate the error-prone correlation, ${\hat{r}}_{xy}$ . We therefore conclude that the quadrature-based method for computing reliability is a better benchmark for the study of reliability estimates that we conduct in the following study.

### Study 2

In order to complement our previous findings, this second study is conducted to compare reliability coefficients with dichotomous items using a simulation study. We focus on reliability coefficients that are popular both historically and currently, as well as include recently developed model-based coefficients. We mainly divide such reliability coefficients into those based on CTT (or observed scores) and those based on a measurement model.

### CTT-Based Coefficients

From Guttman’s (1945) six lower bounds of reliability, $\lambda_{3}$ is the same as the popular Cronbach’s α (1951), which is also a generalization of a coefficient found in Kuder & Richardson (1937). Mathematically,

(13) $$\lambda_{3}=\alpha =\left(\frac{I}{I-1}\right)\left[1-\;\frac{\sum_{i=1}^{I}Var\left(Y_{i}\right)}{Var\left(Y\right)}\right]$$

where $Var\left(Y_{i}\right)$ is the variance of item *i* and Var(Y) is the variance of the sum score composite, *Y*. Under a congeneric model, α is expected to underestimate reliability (e.g., Raykov, 1997), but may also overestimate if errors are correlated (Raykov, 2001). Interestingly, the usefulness of this coefficient was recently discussed with some authors suggesting to abandon α (e.g., Dunn et al., 2013; McNeish, 2018) others argued that this coefficient is still useful (e.g., Raykov & Marcoulides, 2019).

Sijtsma (2009) suggested use of the great lower bound (*GLB*) as an alternative to coefficient α . For the *GLB*, we first present a matrix decomposition of the *I* observed scores for the test (e.g., Jackson & Agunwamba, 1977)

(14) $$\Sigma_{Y}=\Sigma_{T}+\Sigma_{E}$$

where the I×I covariance matrix, $\Sigma_{Y}$ , is decomposed into true score covariances, $\Sigma_{T}$ , and error covariances, $\Sigma_{E}$ , noting that the latter is usually assumed diagonal and contains only error variances. Since the right-hand decomposition of (14) is unknown, the elements of this equation must be estimated. An alternative decomposition is provided by Bentler & Woodward (1980; see also Bentler, 2009) and may be more useful for understanding why the *GLB* is considered a “lower bound” to reliability. The true score covariance matrix is further decomposed, $\Sigma_{T}=\Sigma_{C}+\Sigma_{S}$ , such that

(15) $$\Sigma_{Y}=\Sigma_{C}+\Sigma_{S}+\Sigma_{E}$$

Note that $\Sigma_{C}$ is now the covariance matrix among variables due to some common source of variability and $\Sigma_{S}$ is a matrix that is usually diagonal and contains item-specific variances. In practice, it is easier to think of $\Sigma_{C}$ as covariance among variables that is due to some underlying cause or construct that the test creator is trying to measure.

Finally, the *GLB* is computed as follows

(16) $$GLB=1-\frac{tr\left(\Sigma_{E}\right)\;}{Var\left(Y\right)}$$

where $tr\left(\Sigma_{E}\right)$ is the trace of the inter-item error covariance matrix. In comparing (16) to equation (1), we see that $tr\left(\Sigma_{E}\right)=Var\left(E\right)$ , provided that the matrix is diagonal. Important for understanding this coefficient is that $\Sigma_{S}$ may be considered to contain reliable, though item-specific variance. However, it may be impossible to empirically distinguish between $\Sigma_{S}$ and $\Sigma_{E}$ in a single test administration. Regardless, any estimate of $tr\left(\Sigma_{E}\right)$ may actually be an estimate of $tr\left(\Sigma_{S}+\Sigma_{E}\right)$ , and reliability is arguably underestimated. This occurs since the *GLB* in (16) becomes smaller when the error variances in $\Sigma_{E}$ are larger. The *GLB* is therefore described as the “worse-case scenario” (Sijtsma, 2009; Sijtsma & van der Ark, 2017) as actual computation of (16) is done by maximizing $tr\left(\Sigma_{E}\right)$ subject to the constraints that $\Sigma_{E}$ is nonnegative and $\Sigma_{T}$ is positive semidefinite.

### Model-Based Reliability

Use of a measurement model often entails additional assumptions regarding functional form or the distribution of the variables, but can sometimes offer greater flexibility or additional features (e.g., multidimensional models, adaptive test assembly) if such assumptions are met. In what follows, we will shortly discuss approaches based on both linear and non-linear models.

### Linear Model-Based

Omega ( ω ), is perhaps the most commonly reported linear model-based coefficients and was developed by McDonald (1985, 1999), yet has its roots in work by Joreskog (1971) and is sometimes called coefficient rho, ρ (e.g., Raykov & Shrout, 2002). We use the term “linear” here to indicate that estimates of this reliability coefficient can be derived from parameter estimates of a linear factor analysis model. The common factor model with a single underlying factor takes the following form for item *i*

(17) $$Y_{i}=\mu_{i}+\lambda_{i}\theta +\varepsilon_{i}$$

where $\mu_{i}$ is an intercept, $\lambda_{i}$ is a slope known as a factor loading, θ is a latent trait and $\varepsilon_{i}$ is the random residual of the model (with $Var\left(\varepsilon_{i}\right)=\psi_{i}^{2}$ ). McDonald (1999) equates the variability due to θ as a common source of reliable variance in line with the decomposition in (16)^2^. In the case of a one-factor model and assuming uncorrelated errors, true score (or common) variance is approximated via the sum of squared loadings, $Var\left(T\right)\approx {\left({\sum_{i=1}^{I}\lambda }_{i}\right)}^{2}$ , error variance is approximated by $Var\left(E\right)\approx \sum_{i=1}^{I}\psi_{i}^{2}$ , and results in an estimate of reliability

(18) $$\omega =\frac{{\left(\sum_{i=1}^{I}\lambda_{i}\right)}^{2}}{{\left(\sum_{i=1}^{I}\lambda_{i}\right)}^{2}+\sum_{i=1}^{I}\psi_{i}^{2}}$$

which can be related to the second equality of equation (1), even if there are conceptual differences between both approaches. According to Maydeu-Olivares et al. (2007): “If a good-fitting model can be found, the use of a model based reliability estimate is clearly the best option. For instance, if a one-factor model is found to fit the data well, then the reliability of the test score is given by coefficient ω , and the applied researcher should employ this coefficient” (p. 172). However, we have to mention there are many ways to estimate ω (Revelle & Condon, 2019). In this article, we will focus only on $\omega_{Total}$ .

There are also several ways to estimate $\lambda_{i}$ and $\psi_{i}^{2}$ . By default, the psych package (Revelle, 2019) uses “minres” (ordinary least squares), which we study (ω) in addition to estimation using confirmatory factor analysis estimated via normal theory maximum likelihood (*CFA*). However, this is somewhat theoretically questionable with dichotomously scored data as it may be more reasonable to assume that there is a nonlinear relationship between the underlying common factor and the item responses, which we discuss in the next section.

### Non-Linear Model-Based

We are aware of two general approaches whereby an estimate of reliability is obtained by an IRT or nonlinear factor analysis model. We have already discussed work by Dimitrov (2003a, 2003b) and how true reliability for a test consisting of dichotomous items can be defined in terms of item parameters for the 2PLM. Raykov et al. (2010) took this one step further and reasoned that consistent estimates of item parameters should yield a consistent estimate of reliability. Therefore, it is feasible to estimate all item parameters using the 2PLM and then utilize either analytical (which we call *DA*) or quadrature-based equations (*DQ*) to estimate reliability using Equations (7–11). In our simulations, we take both of these approaches after estimating the 2PLM with the mirt package (Chalmers, 2012) in R.

In addition, Green and Yang (2009b) developed a method based on a nonlinear structural equation model (*GY*). These authors derived an estimate of reliability by defining reliability as the correlation between a test and its parallel form. The resulting equations are in terms pertaining to the item parameter estimates from a nonlinear factor analysis model. In supposing that the latent trait is linearly related to some underlying item response, and that this response variable is dichotomized, the model takes the underlying variable approach. To estimate the model, a limited information estimation approach is employed whereby item thresholds are estimated, followed by polychoric correlations, and then item parameters (see Bolt, 2005). The resulting thresholds and factor loadings can be used to estimate the reliability between a sum score composite of the observed variables and their parallel form, which, in theory, should be the same as classical definitions of reliability. It is well-known that such a factor analysis model of dichotomous items is equivalent to an IRT model such as the 2PLM, except a probit link function is often used in place of a logistic function (Kamata & Bauer, 2008; Takane & De Leeuw, 1987). We therefore speculate that this approach is likely equivalent (or nearly so) to the approach presented by Raykov et al. (2010). Since the equations and derivations for this approach are quite long and require another model parameterization, we refer the reader to the original article for more details (e.g., Green & Yang, 2009b; Equation (21), p. 160).

### Some Theoretical Expectations

By using data generated under a 2PLM, we may get a sense of the behavior of such reliability estimates in relation to realistic slopes and discrimination parameters. We also presume that the most realistic scenario is that item parameters such as the discrimination, $a_{i}$ , and difficulty, $b_{i}$ , vary across items. This has several implications for the expected performance of the just presented reliability estimates.

First, the relationship between the latent trait and the underlying variable is not the same for each variable, and the variance of each variable may be different. In such a case, the so-called congeneric model results (Raykov, 1997). Under this model, coefficient α is expected to be an underestimate of reliability. Although the *GLB* is theoretically a better estimate, some bias and instability has been found in previous research (Sijtsma & van der Ark, 2017).

Next, many authors promote ω because this coefficient was found to be superior to α (e.g., Dunn et al., 2013; Maydeu-Olivares & Hartmann, 2007; McDonald, 1985; Trizano-Hermosilla & Alvarado, 2016). Even though linear model-based estimates of reliability such as ω are derived from what may be considered an incorrect model for the data, to the extent that a linear function of the latent trait still provides a reasonable estimate of the variability due to the trait, ω may be a decent approximation of reliability.

Finally, nonlinear model-based estimates of reliability are theoretically the most correct. However, in finite samples and tests, there may be small differences in both bias and efficiency of item parameter estimates used in IRT and factor analysis of categorical data (e.g., Forero & Maydeu-Olivares, 2009; Rhemtulla et al., 2012), which may lead to small differences in coefficient performance. As test length and sample size increases, however, we would expect that such approaches would perform the best.

In the following simulation study, we compare these coefficients under a variety of sample sizes and test lengths, and vary the true item parameters generated across replications.

## Method

The design crossed 3 test lengths (*I* = 15, 40, 65) and 5 sample sizes (*N* = 100, 300, 500, 1000, 3000). For each combination, 1000 datasets were generated for a total of (3×5)×1000 = 15000 datasets. For each dataset, item parameters were drawn randomly from the following distributions: $\;a_{i}∼logN\left(-.75,.25^{2}\right)$ , $b_{i}∼N\left(0,.75^{2}\right)$ . The latent trait was also normally distributed, θ∼N(0,1) , and such item parameters and latent trait scores were used to generate responses to the dichotomous items using the sim function from the cacIRT R package (Lathrope, 2015).

The coefficients under consideration are presented in the next table (Table 1), and have already been discussed in the previous section, including also *DA* using true item parameters (*DATrue*).^3^ Example code for computing all coefficients appears in Supplementary Materials.

**Table 1.** Coefficients Under Investigation in Study 2

Coefficient name’s	Information	Source
*DATrue*	Method developed by Dimitrov (2003a) based on analytic approximation using the true $a_{i}$ and $b_{i}$ parameters	Custom code in supplementary materials
*DQ*	Method developed by Dimitrov (2003a) based on rectangular quadrature used after estimating item parameters with the 2PLM	Custom code in supplementary materials
*DA*	Method developed by Dimitrov (2003a) based on an analytical approximation used after estimating item parameters with the 2PLM	Custom code in supplementary materials
α	Cronbach’s α or Guttman’s $\lambda_{3}$	alpha function from psych R package Revelle (2019)
*GLB*	*GLB* based on an algebraic procedure	glb.algebraic function from psych R package
ω	$\omega_{Total}$ based on 1 dimension using pearson correlation	omega function from psych R package
*CFA*	Model-based reliability from loadings and errors variances of a single-factor CFA	Custom code using lavaan function from lavaan R package (Rosseel, 2012)
*GY*	Green and yang coefficient (2009b) based on WLS estimator	Custom code using lavaan function from lavaan R package and sirt R package (Robitzsch, 2019)

We compared true reliability to the value of every coefficient by reporting the root mean square error

(19) $$RMSE=\sqrt{\frac{\sum_{s=1}^{1000}{\left({\hat{\rho }}_{s}-\rho_{True}\right)}^{2}}{1000}}$$

and bias

(20) $$Bias=\sum_{s=1}^{1000}\left[\frac{\left({\hat{\rho }}_{s}-\rho_{True}\right)}{1000}\right]$$

where ${\hat{\rho }}_{s}$ is the value of an estimated coefficient (e.g., *DQ*, α or ω ) for simulated dataset *s* and the true reliability $\rho_{True}$ is computed using (10) and (11).

## Results

A similar pattern of results emerged across both sample size and the number of items. We describe these results as concisely as possible, while mentioning general patterns across these manipulated factors.

### RMSE

Not surprisingly, Table 2 shows that RMSE became smaller when the number of items and the sample size increased. The top-three reliability coefficients with the smallest RMSE are *DQ*, *CFA* and ω, respectively, for 15 items; *ω*, *DQ* and *CFA*, respectively, for 40 items; and *ω*, *DQ* and *CFA*, respectively, for 65 items α and *GY* were close behind in both cases. Differences among these approaches appeared negligible, and it was interesting that these coefficients were not very sensitive to sample size.

**Table 2.** RMSE.

*N*	*DATrue*	*DQ*	*DA*	α	*GLB*	ω	*CFA*	*GY*
				15 items				
100	1.338	0.071	1.133	0.179	5.388	0.088	0.020	0.285
300	1.169	0.019	1.071	0.155	2.803	0.035	0.059	0.121
500	1.162	0.044	1.086	0.156	2.163	0.057	0.075	0.202
1000	1.171	0.009	1.159	0.088	1.582	0.004	0.022	0.200
3000	1.174	0.019	1.184	0.064	0.94	0.010	0.005	0.216
				40 items				
100	0.678	0.023	0.587	0.111	3.720	0.022	0.043	0.118
300	0.679	0.007	0.654	0.057	2.424	0.007	0.016	0.055
500	0.682	0.013	0.668	0.046	1.941	0.001	0.011	0.091
1000	0.682	0.001	0.673	0.046	1.141	0.006	0.016	0.123
3000	0.681	0.006	0.673	0.044	0.837	0.007	0.018	0.143
				65 items				
100	0.472	0.007	0.428	0.084	2.922	0.028	0.041	0.074
300	0.469	0.013	0.437	0.042	2.010	0.008	0.015	0.042
500	0.470	0.005	0.448	0.032	1.638	0.003	0.01	0.064
1000	0.470	0.022	0.449	0.034	1.209	0.007	0.014	0.086
3000	0.472	0.017	0.457	0.025	0.732	0.001	0.007	0.096

At the opposite end of performance, *GLB* presented the largest RMSE and appeared to be strongly affected by sample size. Note how the data points for sample size vary greatly for the *GLB* in any given case. For example, in the case of a 15 item test, *GLB* presented an RMSE of 5.388 for *N* = 100 and .94 when *N* = 3000.

### Bias

In line with the previous results, bias (Table 3) became smaller when the number of items and sample size increased. In addition, our results revealed that *DATrue*, *DA* and *GLB* systematically presented a positive bias. That said, the bias was sometimes high, and primarily under conditions with few items. On the other hand, α and *CFA* all presented a systematic negative and small bias over the simulations.

**Table 3.** Bias.

*N*	*DATrue*	*DQ*	*DA*	α	*GLB*	ω	*CFA*	*GY*
				15 items				
100	0.0481	0.0025	0.0408	−0.0065	0.1940	0.0032	−0.0007	0.0103
300	0.0369	−0.0006	0.0339	−0.0049	0.0886	−0.0011	−0.0019	−0.0038
500	0.0367	−0.0014	0.0343	−0.0049	0.0684	−0.0018	−0.0024	−0.0064
1000	0.0370	0.0003	0.0367	−0.0028	0.0500	−0.0001	−0.0007	−0.0063
3000	0.0371	0.0006	0.0375	−0.0020	0.0297	0.0003	−0.0002	−0.0068
				40 items				
100	0.0214	0.0007	0.0186	−0.0035	.11762	−0.0007	−0.0014	0.0037
300	0.0215	0.0002	0.0207	−0.0018	0.0766	−0.0002	−0.0005	−0.0017
500	0.0216	0.0004	0.0211	−0.0014	0.0614	−0.0000	−0.0003	−0.0029
1000	0.0216	0.0000	0.0213	−0.0015	0.0447	−0.0002	−0.0005	−0.0039
3000	0.0215	−0.0002	0.0213	−0.0014	0.0265	−0.0002	−0.0006	−0.0045
				65 items				
100	0.0149	0.0002	0.0136	−0.0026	0.0924	−0.0009	−0.0013	0.0024
300	0.0148	−0.0003	0.0138	−0.0013	0.0636	−0.0003	−0.0005	−0.0013
500	0.0149	−0.0002	0.0142	−0.0010	0.0518	−0.0001	−0.0003	−0.0020
1000	0.0149	−0.0007	0.0142	−0.0011	0.0382	−0.0002	−0.0005	−0.0027
3000	0.0149	−0.0005	0.0145	−0.0008	0.0232	−0.0000	−0.0002	−0.0030

The coefficients with the smallest level of bias were where all model-based coefficients *DQ*, ω and *CFA*. Again, the magnitude of bias did not seem to be affected by the sample size or the number of items. At the opposite end, *GLB* coefficients were those with the largest bias, which is particularly true when the sample size decreased and may explain part of the reason *GLB* coefficients had unstable RMSE.

## Discussion

Several coefficients performed well in our simulation studies. Model-based coefficients *DQ*, ω and *CFA* presented the smallest RMSE and bias. Any of these would be a good choice if the user wishes to choose a model-based reliability coefficient, though these may require (testable) model assumptions. Generally speaking, although presenting a single coefficient based on an IRT model can be criticized, *DQ* may provide a reasonable estimate and future research may show it is a good alternative to so-called Person separation indices (e.g., see Mair et al., 2018; Rasch, 1960).

The *GLB* was strongly affected by the sample size and is not recommended for a unidimensional test. This is in accordance with Shapiro & Ten Berge (2000). In addition, we found a positive bias as also observed by Ten Berge & Sočan (2004) for this coefficient.

Recent critiques against Cronbach’s α restored the interest of a larger audience for reliability (Dunn, Baguley & Brunsden, 2013; Green & Yang, 2009a; McNeish, 2018; Revelle & Zinbarg, 2009; Sijtsma, 2009; Trizano-Hermosilla & Alvarado, 2016). If a model-based coefficient such as *DQ*, ω and *CFA* can be computed, this is preferred. α may be a reasonable alternative only if there are software limitations or if estimation of a model proves difficult (e.g., in situations where the number of items is large or participants are few). Model-based coefficients are more precise and do not require tau-equivalent measurement model assumptions.

## Conclusion

This article integrates 2 studies. First, we found that Dimitrov’s (2003a, 2003b) strategy based on quadrature is a better benchmark for the study of reliability. Second, we used a simulation study to analyze the precision of some CTT-based and model-based reliability coefficients. Our results show that Dimitrov’s (2003a, 2003b) quadrature-based strategy, ω based on a single dimension, and *CFA*-based on a single dimension are all the most precise coefficients. At the opposite, *GLB* is the least interesting coefficient among those under investigation.

This article is obviously not without limitations. As many coefficients studied here have not been previously compared, it was logical to first study them under ideal conditions (i.e., a correctly specified model). It is important in future work to study them under assumption violations (multiple dimensions and locally dependent items; see also van der Ark, van der Palm, & Sijtsma, 2011) and more complex models to understand how well these coefficients behave and where such reliability coefficients may result in larger discrepancies.

## Notes

1. We are however aware that many authors criticize the use of sum scores (e.g. Kuhfeld & Soland, 2020; McNeish & Wolf, 2020)

2. On p. 88 of McDonald (1999), for example, the notation “C” is used in place of “T” to indicate common factor, and “U” instead of “E” to indicate a unique part of the item in (1).

3. Several other reliability coefficients were studied for exploratory purposes, but did not perform well or were not as theoretically justified and are omitted due to space constraints: Other variants of Guttman’s coefficient, *GLB* estimated using factor analytic approach, defaults for $\omega_{Total}$ including three extracted factors or use of polychoric correlations. Results are available upon request.
